# Comparative Transcriptional Profiling of Motor Neuron Disorder-Associated Genes in Various Human Cell Culture Models

**DOI:** 10.3389/fcell.2020.544043

**Published:** 2020-09-24

**Authors:** Stefan Hauser, Stefanie Schuster, Elena Heuten, Philip Höflinger, Jakob Admard, Yvonne Schelling, Ana Velic, Boris Macek, Stephan Ossowski, Ludger Schöls

**Affiliations:** ^1^German Center for Neurodegenerative Diseases (DZNE), Tübingen, Germany; ^2^Department of Neurology and Hertie Institute for Clinical Brain Research, University of Tübingen, Tübingen, Germany; ^3^Graduate School of Cellular and Molecular Neuroscience, University of Tübingen, Tübingen, Germany; ^4^Institute of Medical Genetics and Applied Genomics, University of Tübingen, Tübingen, Germany; ^5^Proteome Center Tübingen, University of Tübingen, Tübingen, Germany; ^6^Center of Rare Diseases, University of Tübingen, Tübingen, Germany

**Keywords:** motor neuron disorders, hereditary spastic paraplegia, amyotrophic lateral sclerosis, spinal muscular atrophy, gene expression, iPSCs, neurons, disease modeling

## Abstract

Disease modeling requires appropriate cellular models that best mimic the underlying pathophysiology. Human origin and an adequate expression of the disease protein are pre-requisites that support information from a model to be meaningful. In this study we investigated expression profiles of (i) PBMCs and (ii) fibroblasts as patient derived cells as well as (iii) lymphoblasts and (iv) induced pluripotent stem cells (iPSC) as immortalized sources, and (v) iPSC-derived cortical neurons to assess their aptitude to model motor neuron diseases (MNDs) including hereditary spastic paraplegia (HSP), amyotrophic lateral sclerosis (ALS) and spinal muscular atrophy (SMA). We generated all five different cell types from two healthy donors and performed RNA sequencing to display expression patterns in MND-related genes. For the ten most common HSP genotypes we validated gene expression by qPCR. To verify the results on protein level, proteome analysis of fibroblasts, iPSCs and cortical neurons was performed. Depending on the specific MND gene we found largely different expression patterns. Out of 168 MND-related genes, 50 had their highest expression in iPSC-derived cortical neurons, 41 were most strongly expressed in fibroblasts, 26 in lymphoblasts, 22 in iPSCs, and 14 in PBMCs. Pathophysiologically related MNDs like HSPs associated with axonal transport deficits shared highest expression in cortical neurons. 15 MND-related genes were not detectable in any of the analyzed cell types. This may reflect the critical dependency of motor neurons on support of other cell types like oligodendrocytes which express myelin proteins like L1CAM (SPG1), PLP1 (SPG2) and MAG (SPG75) which are lacking in neurons but cause MNDs if mutated. This study provides comprehensive information on expression of genes associated with a large spectrum of MNDs. Expression profiles can be used to inform on appropriate cell models for genotype specific motor neuron research.

## Introduction

Studies on pathophysiology of human disease and the development of new therapeutics are essentially dependent on suitable disease models. This is particularly difficult in neurological disease as often only the nervous system is affected and access to human neuronal tissue is limited to nerve and brain biopsies or post-mortem material that are notoriously rare with very limited access.

Primary patient cells like peripheral blood mononuclear cells (PBMCs) isolated from blood or fibroblasts isolated from skin biopsies are easily accessible but not primarily affected by the disease and therefore might represent pathophysiological processes only to a limited and often unknown extent. Additionally, primary patient cells possess limited growth capacity which hampers their use in large-scale screening approaches. To overcome this limitation strategies of immortalizing primary patient cells have been developed early on, e.g., the transformation of PBMCs (mainly B lymphocytes) to lymphoblasts by using the Epstein-Barr virus (Miller, 1982). With the discovery of induced pluripotent stem cells (iPSCs) a novel and promising technique to generate disease-relevant and patient-specific cell types arose (Takahashi and Yamanaka, 2006). Over the last years iPSCs became a widespread tool to generate neuronal cell types that are genetically identical with patients suffering from the neurological disease of interest. iPSCs can be reprogrammed from easily accessible cell types like fibroblasts, PBMCs or keratinocytes according to well standardized procedures (Okita et al., 2013; Hauser et al., 2016a). iPSC-derived neurons have been used to model many human neurological diseases including motor neuron diseases (MNDs).

MNDs comprise three major groups of rare neurodegenerative diseases including (i) hereditary spastic paraplegia (HSP) involving primarily upper motor neurons, (ii) spinal muscular atrophy (SMA) with lower motor neuron affection, and (iii) amyotrophic lateral sclerosis (ALS) with rapid degeneration of upper and lower motor neurons. All three MNDs are genetically highly heterogeneous and split up into more than 80 genotypes of HSP, at least 30 genes causing ALS and 18 genes associated with either proximal or distal SMA (Prior et al., 1993; Kinsley and Siddique, 2015; Hedera, 2018). Further, MNDs show a substantial overlap with other disease groups like cerebellar ataxias, leukoencephalopathies and hereditary peripheral neuropathies (Synofzik and Schüle, 2017). In total more than 160 genes related to motor neuron impairment are known to date.

Until the discovery of iPSCs, research on MND mostly focused on mouse models and cell culture models using immortalized murine and human cell lines. Animal models are advantageous in providing an *in vivo* system with its whole complexity but differences between species are huge and transferability to human disease is difficult. This is especially true in the context of neurological disorders. The hope that mouse models mimic human disease pathology has been disappointing in particular for MNDs since most mouse models did not develop motor function defects or preclinical drug trials in mouse models were not successfully transferable to human studies (reviewed in Genc et al., 2019).

The potential of iPSC to generate a patient-specific cell model that represents the cell type affected by the disease of interest with the same genetic background opened new possibilities to study MND in a human neuronal cell culture model (reviewed in Denton et al., 2016). For example, analysis of SPAST-deficient neurons generated from SPG4 patients revealed pathophysiological axonal defects like axonal swellings, a hallmark of HSP disease pathology, in human iPSC-derived neurons (Havlicek et al., 2013; Denton et al., 2014). Further, in a study of SPG5, iPSC-derived neurons were employed to decipher the neurotoxic role of oxysterols that is likely to drive pathogenesis in this subtype of HSP (Schöls et al., 2017). Additionally, iPSC-derived neurons have been used for pharmacological screens to identify novel targets for the treatment of MNDs e.g., in SPG4 and SPG11 (Pozner et al., 2018; Rehbach et al., 2019). iPSC-derived motor neurons and cortical neurons have also been used to mimic familial as well as sporadic forms of ALS. Observed phenotypes range from e.g., cytoskeletal defects, impaired axonal transport, cell death and vulnerability, ER stress and mitochondrial dysfunction (reviewed in Guo et al., 2017).

To choose an adequate disease model is of utmost importance for medical research. The presence of disease-related molecules is a pre-requisite of meaningful cell models. Using RNA-Sequencing (RNA-Seq) analyses we here compared the transcript expression profiles of more than 160 MND-related genes including all spastic paraplegia genes (SPGs), SMA genes and ALS genes known so far in biosamples of two independent healthy individuals including (i) PBMCs and (ii) fibroblasts as primary cells, (iii) lymphoblasts and (iv) iPSCs as immortalized cell lines as well as, and (v) iPSC-derived cortical projection neurons representing a cell type predominantly affected in HSP and ALS. This dataset may help to inform about the ideal source of a patient-specific human cell culture that endogenously expresses the gene of interest to model MNDs.

## Materials and Methods

### Isolation of Primary Cells

The study has been approved by the Ethics Committee of the University of Tübingen (598/2011BO1). Informed consent was obtained from all participants.

Fibroblasts were obtained from skin biopsies of two healthy individuals (Donor 1: age: 46, gender: female/Donor 2: age: 37 gender: female). Skin tissue was dissected and cultivated in a 25 cm^2^ tissue culture flask containing Dulbecco’s modified eagle’s medium (DMEM) high glucose (Life technologies) with 10% fetal bovine serum (FBS, Life technologies) (fibroblast medium) for 10 days at 37°C and 5% CO_2_. Fibroblast expansion was achieved by medium change every 2–3 days.

PBMCs were isolated from whole blood of the same healthy donors as fibroblasts using a classical density gradient centrifugation with Ficoll (Ficoll Paque-PLUS, GE Healthcare) according to manufacturer’s guidelines.

### Transformation of PBMCs to Lymphoblasts

Immortalized lymphoblast cell lines were generated by infection of PBMCs (mainly B lymphocytes) using the EBV (Pelloquin et al., 1986). In brief, separated PBMCs were resuspended in 2 ml sterile EBV-containing supernatant of B95-8 cells and incubated for 30 min at 40°C. EBV-transformed lymphocytes (lymphoblasts) were cultivated in RPMI medium (Sigma-Aldrich) containing 20% FBS, 1% Penicillin/Streptomycin (Biochrom), 1 μg/ml Cyclosporin A (Sigma-Aldrich), 1 mM sodium pyruvate (Sigma-Aldrich), 20 mM HEPES (Sigma-Aldrich) and 1% L-glutamine (Merck). Lymphoblasts were frozen in cryopreservation medium containing of 90% FBS supplemented with 10% DMSO (Sigma-Aldrich).

### Reprogramming of Fibroblasts to iPSCs

Reprogramming was achieved by electroporation of 1 × 10^5^ fibroblasts with 1 μg of episomal plasmids (pCXLE-hUL, pCXLE-hSK, and pCXLE-hOCT4) as described by Okita et al. (2011). 1 day after electroporation fibroblast growth factor-2 (FGF-2, 2 ng/mL, Peprotech) was supplemented to the fibroblast media. The following day cells were cultivated in Essential 8 (E8) medium supplemented with 100 μM sodium butyrate (Sigma-Aldrich) with media change every other day. Approx. 3–4 weeks after electroporation iPSC colonies were manually picked and further expanded on Matrigel-coated well plates. Splitting and replating of iPSCs was achieved by adding PBS/EDTA (0.02% EDTA in PBS). iPSCs were genomically and functionally analyzed according to (Hauser et al., 2016b).

### Differentiation of iPSCs to Cortical Neurons

Induced pluripotent stem cells were differentiated to neurons of cortical layer V and VI according to published protocols with minor modifications (Shi et al., 2012; Rehbach et al., 2019). Briefly, iPSCs were seeded at a density of 3 × 10^5^ cells per cm^2^ in E8 medium supplemented with 10 μM Y-27632 (Selleckchem). Neural induction was obtained by supplementation of SMAD inhibitors [10 μM SB431542 (Sigma-Aldrich) and 500 nM LDN-193189 (Sigma-Aldrich)] to 3N medium for 10 days. On day 10, cells were split in a 1:3 ratio and further expanded in 3N medium including 20 ng/ml FGF-2 for 2 days. Until day 27, cells were cultivated in 3N medium with media change every other day. On day 27, cells were dissociated using Accutase (Sigma-Aldrich) and replated at a density of ∼8 × 10^5^ cells per cm^2^. The following day, 10 μM PD0325901 (Tocris) and 10 μM DAPT (Sigma Aldrich) was added to 3N medium with an additional media change at day 30. From day 32 onward 3N medium was changed every other day and RNA of cortical neurons was isolated at day 37 of differentiation.

### Immunocytochemistry

Cells were fixed with 4% paraformaldehyde (PFA, Merck Millipore) for 15 min and subsequently washed with PBS. After permeabilization and blocking with 5% BSA (Sigma Aldrich) and 0.1% Triton X-100 (Carl Roth) in PBS, cells were stained with: anti-Vimentin (rabbit, 1:100, 5741S, CST), anti-OCT4 (rabbit, 1:100, 11263-1-AP, Proteintech), anti-TRA1-81 (mouse, 1:500, MAB4381, Merck Millipore), anti-ß-III-tubulin (TUJ, mouse, 1:1000, T8660, Sigma Aldrich), anti-TBR1 (rabbit, 1:200, ab31940, Abcam) and/or anti-CTIP2 (rat, 1:200, ab18465, Abcam). After an additional washing step, Alexa-Fluor conjugated secondary antibodies (Invitrogen) were applied. Nuclei were stained with Hoechst 33258 staining (1:10.000, H1398, Invitrogen). Coverslips were mounted with Dako Mounting Solution (Agilent Dako) and images acquired using Zeiss Observer Z1 fluorescence microscope (Carl Zeiss).

### RNA-Sequencing Analysis

RNA isolation of cells (PBMCs, lymphoblasts, fibroblasts, iPSCs, iPSC-derived cortical neurons) was performed by using the RNeasy Mini Kit (Qiagen) according to the manufacturer’s guidelines. Further purification of isolated RNA was achieved with TruSeq mRNA v2Kit (polyA) (Illumina). RNA samples (1 sample per donor and cell type) were sequenced on a HiSeq2500 (Illumina) by DeCODE genetics (Reykjavik, Iceland).

For further expression analysis and to calculate the sample similarity a multi-dimensional scaling (MDS) plot of all RNA-Seq samples was prepared. For comparison of 168 MND-related genes, RPKMs (Reads Per Kilobase Million) for each transcript and cell type were calculated.

For GO term analysis the total number of genes with a mean expression >3 RPKMs was determined for each Gene Ontology category and cell type, resulting in a GO term coverage metric for MND-related gene sets.

For single cell RNA sequencing comparison TPM (Transcripts Per Kilobase Million) normalized gene expression values of cells and reference scRNA samples were used for pairwise comparisons to calculate Spearman’s rank correlation coefficients as a measure for sample similarity. Wilcoxon’s rank-sum test was applied on correlation coefficients to determine significantly higher similarity values than the background of all comparisons. Heatmaps were used to show significances as –log 10 *p*-values.

All images were generated by using GraphPad Prism.

### qRT-PCR Validation

500 ng isolated RNA of each sample was reverse-transcribed to cDNA by using the RevertAid First Strand cDNA Synthesis Kit (Thermo Fisher Scientific) according to manufacturer’s instructions. Real-time qPCR was performed in triplicates by adding 2 μl primer pairs (2 μM) and 5 μl SYBR Green Select Master Mix (Applied Biosystems) to 3 μl cDNA (1.25 ng/μl).

For amplification the following primers were used:

**Table:** 

Primer	Forward sequence	Reverse sequence
ATL-1/SPG3	CAGTCCAAGTCCTCATTGTC	AATCAACTGATTCCTGGTTGTA
BSCL2/SPG17	TTCTACTACAGGACCGACTG	CAGCTCAAGCTCTAAGGTAAC
CYP7B1/SPG5	CAGTTCTTCTTGGTGGAAAGTA	TGCAACTGACTGATGCTAAA
FA2H/SPG35	TGGGAGAGAAGTACGATGAG	GGGACACTGTACCAGACA
GAPDH	TCACCAGGGCTGCTTTTAAC	GACAAGCTTCCCGTTCTCAG
KIF5A/SPG10	GTCACCAACATGAATGAACAC	CCAGGTCCACCAGATACA
REEP1/SPG31	CCTTTGTTGGTTTCCATTCTAT	CAGACAATCATCGATTTCCTTT
SPAST/SPG4	ATAGTTACAGGACAAGGTGAAC	ACGTCCGTTTGTGACTTG
SPG7/SPG7	CAGGATTCTTTGGAAATGCC	CCATCTTCCCATCCACAAT
SPG11/SPG11	GTATTTCAGGCAACACCCA	GGCTTTCCAAGACCTATCAAT
TBP	CTTCGGAGAGTTCTGGGATTG	CACGAAGTGCAATGGTCTTTAG
ZFYVE26/SPG15	ATACAGCAGAGCAGCAAC	TCTGTAAGAGCTTGAGAACATC

Real-time qPCR was performed on the Viia7 Real-Time PCR System (Applied Biosystems) applying the following qRT-PCR program: 50°C for 2 min, 95°C for 2 min, 40 cycles of 95°C for 1 s, 60°C for 30 s and 72°C for 5 s, followed by 95°C for 15 s, 60°C for 1 min and 95°C for 15 s. Melting curve analysis confirmed the specificity of the PCR products. Further analysis was performed with QuantStudio Software V1.3 (Thermo Fisher Scientific) and visualized and statistically analyzed using GraphPad Prism. For statistical analysis One-way ANOVA was applied. *P*-values were corrected for multiple comparisons and *P* < 0.05 was considered statistically significant.

### NanoLC-MS/MS Analysis and Data Processing

For mass spectrometry pellets of fibroblasts, iPSCs and CNs were lysed for 45 min at 4°C in RIPA buffer (Sigma Aldrich) including 1× cOmplete protease inhibitor cocktail (PI) (Roche). Protein concentration was determined using the Pierce BCA Protein Assay Kit (Thermo Fisher Scientific) according to manufacturer’s guidelines.

Protein extracts were purified using SDS PAGE (Invitrogen). Coomassie-stained gel pieces were excised and in-gel digested using trypsin as described previously (Borchert et al., 2010). After desalting using C18 Stage tips (Rappsilber et al., 2007) extracted peptides were separated on an Easy-nLC 1200 system coupled to a Q Exactive HF mass spectrometer (both Thermo Fisher Scientific) as described elsewhere (Kliza et al., 2017) with slight modifications: The peptide mixtures were separated using a 57 min segmented gradient from to 10-33-50-90% of HPLC solvent B (80% acetonitrile in 0.1% formic acid) in HPLC solvent A (0.1% formic acid) at a flow rate of 200 nl/min. The 12 most intense precursor ions were sequentially fragmented in each scan cycle using higher energy collisional dissociation (HCD) fragmentation. Each samples was measured as technical triplicate.

Acquired MS spectra were processed with MaxQuant software package version 1.6.7.0 (Cox and Mann, 2008) with integrated Andromeda search engine (Cox et al., 2011). MS/MS spectra were searched against a target-decoy Uniprot database consisting of 95972 protein entries from Homo sapiens and 286 commonly observed contaminants. Database search was performed against a target-decoy Roseburia hominis database obtained from Uniprot, containing 11,460 protein entries and 286 commonly observed contaminants. Endoprotease trypsin was defined as protease with a maximum of two missed cleavages. Oxidation of methionine and N-terminal acetylation were specified as variable modifications, whereas carbamidomethylation on cysteine was set as fixed modification. Initial maximum allowed mass tolerance was set to 4.5 parts per million (ppm) for precursor ions and 20 ppm for fragment ions. Peptide, protein and modification site identifications were reported at a false discovery rate (FDR) of 0.01, estimated by the target/decoy approach (Elias and Gygi). The iBAQ (Intensity Based Absolute Quantification) and LFQ (Label-Free Quantification) algorithms as was the ‘match between runs’ option were enabled (Luber et al., 2010; Schwanhausser et al., 2011).

## Results

**FIGURE 1:**
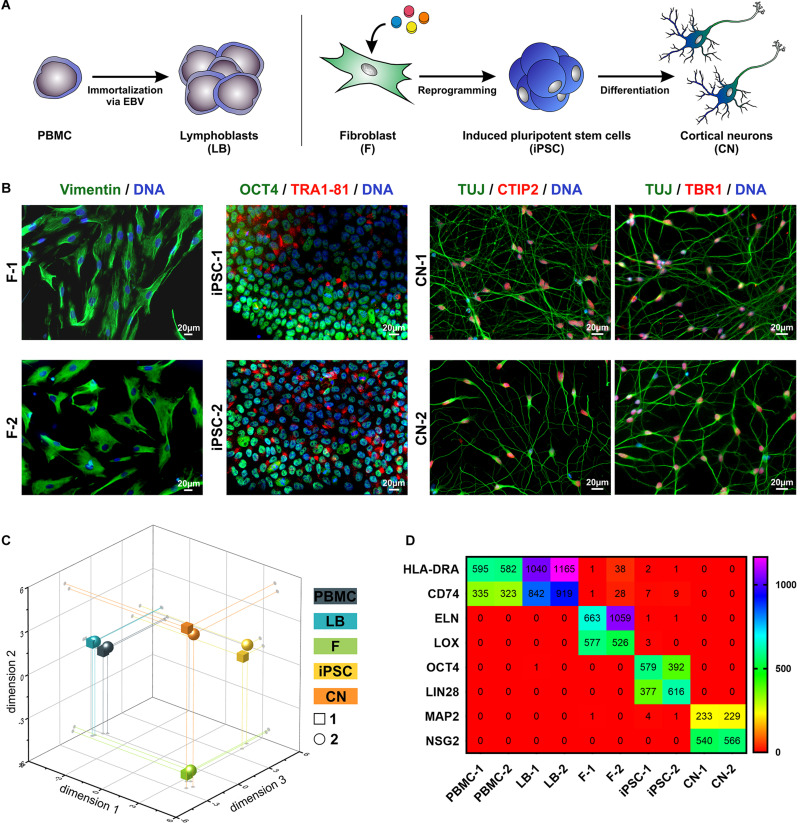
Overview of patient-relevant cell types used for RNA-Sequencing profiling. **(A)** Peripheral blood mononuclear cells (PBMC) were isolated from whole blood and immortalized to lymphoblasts (LB) by EBV (Epstein-Barr Virus)-transformation. Fibroblasts (F) were isolated from skin biopsies, reprogrammed to induced pluripotent stem cells (iPSC) and further differentiated to cortical neurons (CN). **(B)** Immuncytochemical stainings of F (Vimentin), iPSC (OCT4, TRA1-81) and CN (TUJ, CTIP2, and TBR1). Scale bar = 20 μm. **(C)** Multidimensional scaling plot (MDS, 3-dimensional) of all analyzed RNA-Seq samples. **(D)** RPKM (reads per kilobase million) values of cell type-specific markers of the two healthy donors (1 and 2).

To analyze MND-related gene expression in different cell types of the same individual PBMC as well as fibroblasts (F) from two healthy donors were isolated from whole blood and skin biopsies. An immortalized lymphoblast cell line (LB) was generated by infection of PBMCs using the Epstein-Barr Virus (EBV). Fibroblasts were reprogrammed to iPSCs and further differentiated to cortical projection neurons (CN) (Figure 1A). By this, we were able to compare various cell types ranging from easily accessible primary cells over immortalized cell lines to cell types affected by MNDs. The identity of fibroblasts, iPSCs and CNs was further validated by immunocytochemical stainings of typical markers (Figure 1B). Fibroblasts showed typical morphology and expressed Vimentin, iPSC were characterized by expression of OCT4 and TRA1-81 and iPSC-derived cortical neurons of layer V/VI were validated by 100% TUJ- and >85% CTIP2/TBR1-positive cells.

By comparing the global RNA-Sequencing profile of the various cell types we found a highly different and cell type-specific gene expression pattern (Figure 1C). As expected PBMCs and lymphoblasts closely clustered together while the other analyzed cell types showed very divergent and unique global transcript profiles as shown by principal component analysis. Of interest, there was high similarity between the same cell types of different individuals. This is also represented by the expression of cell type-specific markers (Figure 1D). PBMCs and lymphoblasts exclusively expressed the marker HLA-DRA (HLA class II histocompatibility antigen, DR alpha chain) and CD74 (HLA class II histocompatibility antigen gamma chain), while fibroblast could be discriminated by expression of ELN (elastin) and LOX (lysyl oxidase). Pluripotency markers like OCT4 (octamer-binding transcription factor 4) and LIN28 (lin-28 homolog A) were exclusively detectable in iPSCs, whereas MAP2 (microtubule-associated protein 2) and NSG2 (neuronal vesicle trafficking-associated protein 2) were highly expressed solely in iPSC-derived cortical neurons. This highlights the purity of our cell culture systems and supports the validity of this analysis.

To compare the gene expression profile in the different cell types a list of 168 MND-related genes was generated. This list covered all HSP genes (SPGs) known at the time of study including 65 HSP genes (SPG1 – SPG80; SPGs with defined chromosomal loci but unknown genes could not be analyzed), 30 ALS-related genes, 18 SMA-related genes and 55 spasticity-related genes. To normalize for sequencing depth we divided the total reads per sample by 1,000,000 (RPM – reads per million) and further normalized for gene length by dividing the RPM values of each gene by the length of the gene in kilobases (RPKM – reads per kilobase million).

**FIGURE 2:**
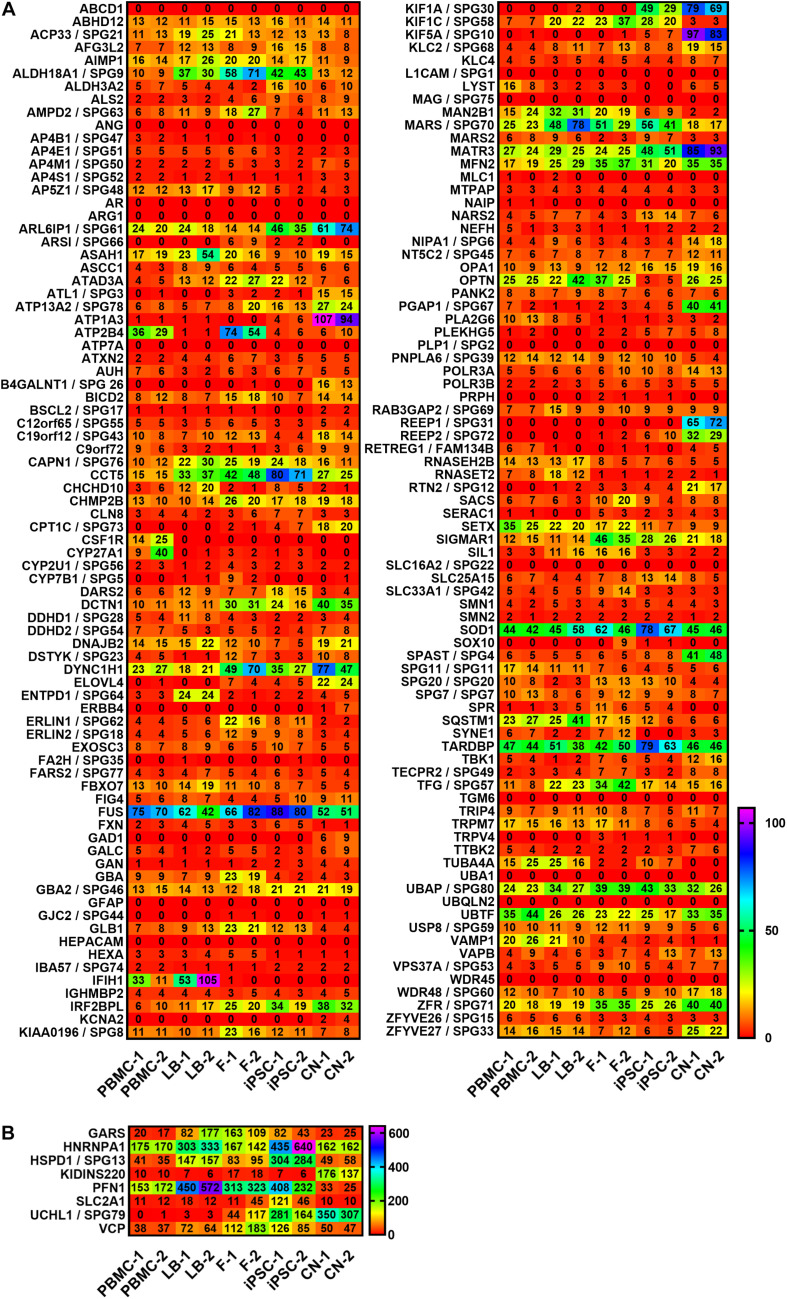
Expression profiling of MND-related transcripts in RPKMs (reads per kilobase million) in five cell types derived from healthy donors (1 and 2). Alphabetic order of MND-related genes with **(A)** RPKMs ranging from 0 to 105 and with **(B)** RPKMs with at least one value >105. HSP transcripts are encoded as: gene name/SPG type. Other MND-related transcripts are encoded as: gene name. RPKMs below 0.5 were set as 0. PBMC, peripheral blood mononuclear cells; LB, lymphoblasts; F, fibroblasts; iPSC, induced pluripotent stem cells; CN, iPSC-derived cortical neurons.

A summary of all analyzed MND-related genes in an alphabetic order can be seen in Figure 2. RPKM values ranged from 0 to 538 depending on cell type and gene of interest. For a better visualization, genes with RPKMs >105 were separately visualized (Figure 2B). We observed high variability in gene expression between cell types of the same donor and low variability between cells of the same type from different donors.

**FIGURE 3:**
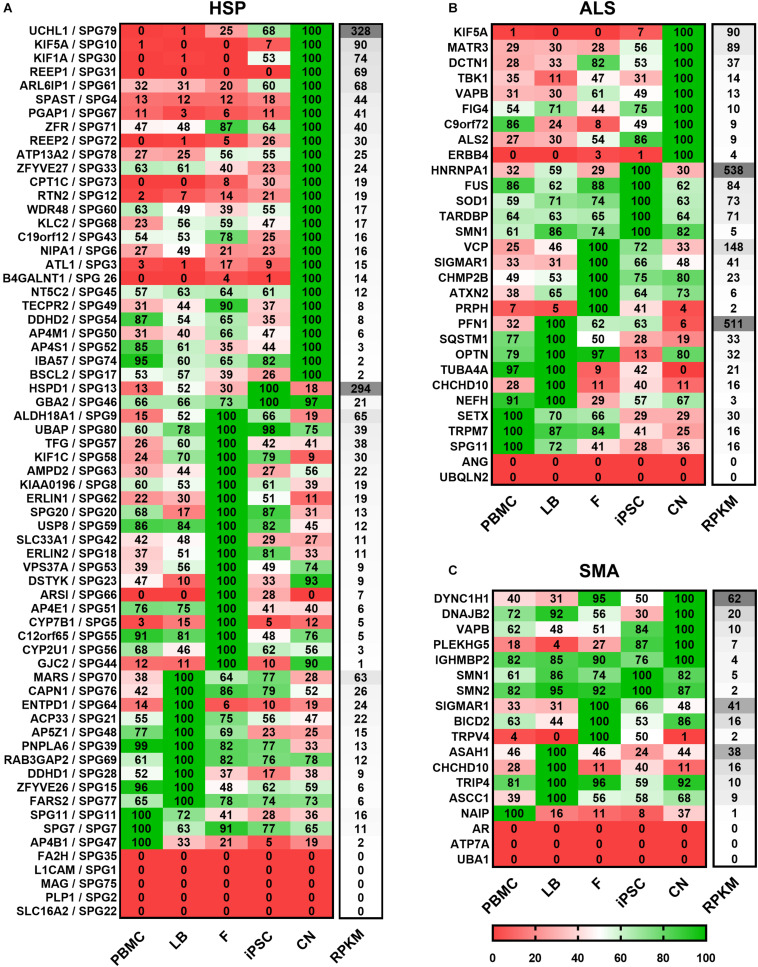
Expression of **(A)** HSP-, **(B)** ALS-, and **(C)** SMA-related transcripts classified according to the cell type showing the highest expression. Mean value of two donors were calculated for each gene, cell type and disease and the highest value of each gene was set to 100%. For each cell type relative values (in%) are given for the respective gene. Grouping per cell type is shown from high to low RPKMs. HSP transcripts are encoded as: gene name/SPG type. Other MND-related transcripts are encoded as: gene name. PBMC, peripheral blood mononuclear cells; LB, lymphoblasts; F, fibroblasts; iPSC, induced pluripotent stem cells; CN, iPSC-derived cortical neurons; RPKMs, reads per kilobase million.

For a clearer presentation and easier comparison of the different gene expression patterns the relative expression values for each gene and disease were determined. Therefore, the mean values of both donors per gene and cell type were calculated and the highest value of each gene was set to 100% (Figure 3). Afterward genes were classified per disease (HSP, ALS, and SMA) and grouped according to the highest expression per cell type from high to low RPKMs.

Out of 65 HSP-related genes, 26 had their highest expression in iPSC-derived cortical neurons ranging from 2 to 328 RPKMs. two were most strongly expressed in iPSCs (range: 21 – 294 RPKMs), 19 in fibroblasts (range: 1 – 65 RPKMs), 10 in lymphoblasts (range: 6 – 63 RPKMs), and 3 in PBMCs (range: 2 – 16 RPKMs). 5 of 65 HSP-related genes were not detectable in any of the analyzed cell types.

Concerning the 30 ALS-causing genes, 9 were expressed highest in neurons (range: 4 – 90 RPKMs) whereas, 5 showed higher expression in iPSCs (range: 5 – 538 RPKMs), 5 in fibroblasts (range: 2 – 148 RPKMs), 6 in lymphoblasts (range: 3 – 511 RPKMs) and 3 in PBMCs (range: 16 – 30 RPKMs). ANG and UBQLN2 were not detectable at all.

Out of the 18 analyzed SMA-related genes, 5 were most abundant in neurons (range: 4 – 62 RPKMs), 2 in iPSCs (range: 2 – 5 RPKMs), 3 in fibroblasts (range: 2 – 41 RPKMs), 4 in lymphoblasts (range: 9 – 38 RPKMs), 1 in PBMCs (1 RPKM). 3 genes (AR, ATP7A and UBA1) were not detected in any of these cell types.

**FIGURE 4:**
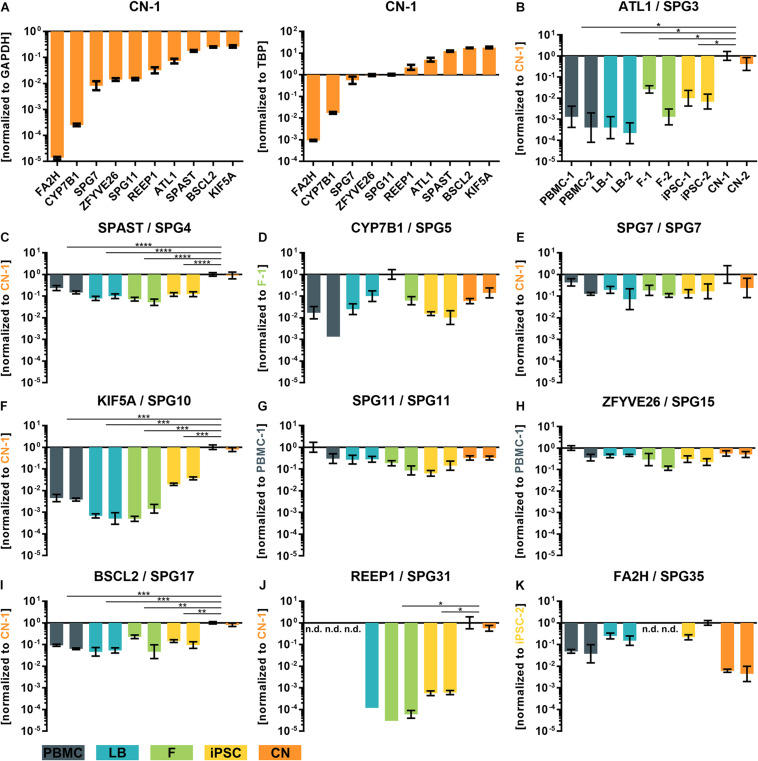
qRT-PCR validation of transcripts of the 10 most common subtypes of HSP. **(A)** ΔCT-values of iPSC-derived cortical neurons of donor 1 (CN-1) normalized to the housekeeping genes GAPDH and TBP. **(B–K)** Relative values (ΔΔCT) of all analyzed cell types and transcripts normalized to the housekeeping genes GAPDH and TBP and to the cell line with highest transcript expression value (set to 1). *: *P* < 0.05; **: *P* < 0.01; ***: *P* < 0.001; ****: *P* < 0.0001; One-way ANOVA. nd, not detectable; PBMC, peripheral blood mononuclear cells; LB, lymphoblasts; F, fibroblasts; iPSC, induced pluripotent stem cells; CN, iPSC-derived cortical neurons.

To validate and replicate the results of the RNA-Seq analysis, the 10 most common HSP genes according to a large representative HSP cohort (Schüle et al., 2016) were selected for further qRT-PCR determination and validation (Figure 4). Two of these genes (KIF5A and SPG11) are also associated with ALS. In general, expression profiles were found to be largely similar in RNA-Seq data and qRT-PCR for SPG3 (ATL1), SPG4 (SPAST), SPG5 (CYP7B1), SPG7 (SPG7), SPG10 (KIF5A), SPG11 (SPG11), SPG15 (ZFYVE26), SPG17 (BSCL2), SPG31 (REEP1), and SPG35 (FA2H) (Figures 4B–K).

For example, REEP1 was almost exclusively expressed in iPSC-derived cortical neurons (CNs) while SPAST was approximately 10 times lower expressed in PBMCs, LBs, Fs and iPSCs compared to CNs. Again, a very low variability of qRT-PCR data was observed between the same cell types of two independent donors.

**FIGURE 5:**
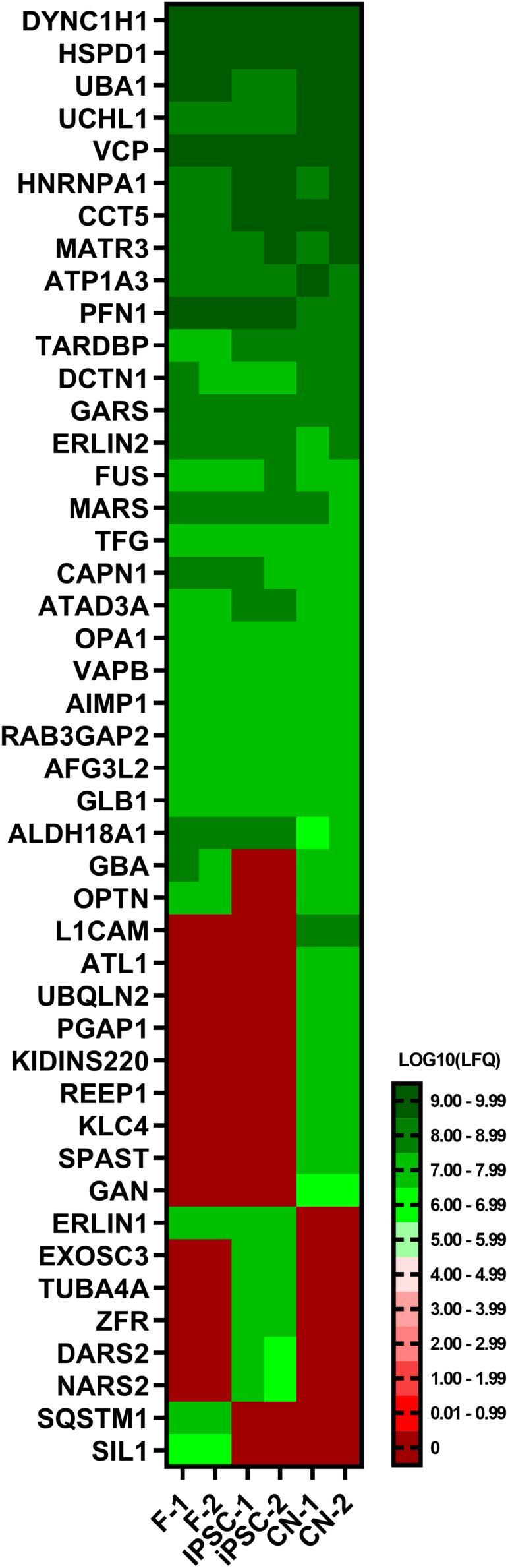
Proteome analysis of fibroblasts, iPSCs and cortical neurons. Heatmap of untargeted proteome analysis in fibroblasts, iPSCs and cortical neurons displaying consistently identified MND-proteins. Mean values of technical triplicates are given as LOG10 label-free quantification (LFQ) intensity.

To further validate the results of the RNA-Seq analysis, a comparative proteomic analysis of fibroblasts, iPSCs and CNs was performed. In total, 45 out of 168 MND-related proteins were identified via an untargeted proteomics approach (Figure 5). Overall, protein expression nicely matches the RNA-Seq results. For example, ATL1, REEP1, and SPAST could only be identified in iPSC-derived cortical neurons while TUBA4A was exclusively expressed in iPSCs and SIL1 was only detectable in fibroblasts.

**FIGURE 6:**
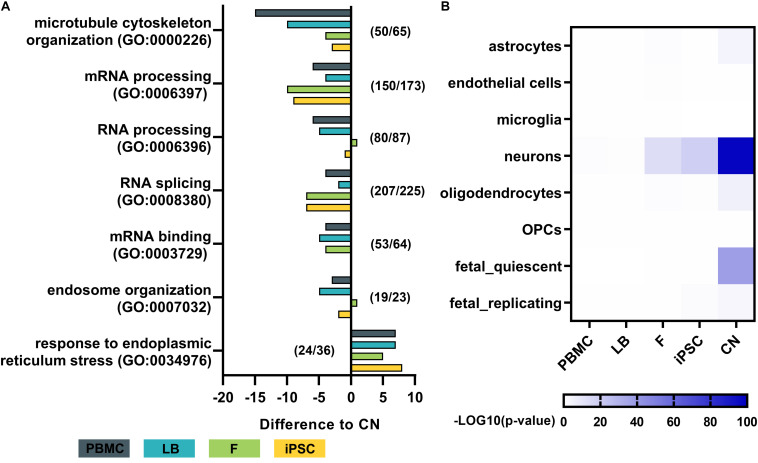
Pathway analysis and dataset comparison of RNA-Seq results. **(A)** Gene ontology (GO) term analysis for each cell type of major disease-related pathways. The total number of genes with a mean expression >3 RPKMs was determined and normalized to the total number of genes per GO term in cortical neurons. Numbers (e.g., 50/65) indicate the number of genes identified in cortical neurons (50) out of the total number of genes per GO term (65). **(B)** Heatmap by Wilcoxon’s rank sum comparison of RNA-Seq dataset to a single cell RNA-Seq dataset of human cortical samples (Darmanis et al., 2015). Values are shown as –LOG10 *p*-values of significant differences. Bulk RNA-Seq of iPSC-derived cortical neurons display a high similarity to human adult neurons of the cortex. PBMC, peripheral blood mononuclear cells; LB, lymphoblasts; F, fibroblasts; iPSC, induced pluripotent stem cells; CN, iPSC-derived cortical neurons.

To analyze the effect of different cell types on the expression of disease-related genes of a specific pathway the expression profiles of major downstream pathological pathways like RNA-processing abnormalities, microtubule cytoskeleton organization, ER stress response, and endosome organization were assessed. For each cell type the total numbers of genes expressed in a specific Gene Ontology category was calculated, resulting in a GO term coverage metric for selected gene sets (Figure 6A). Genes with mean expression of at least 3 RPKMs were labeled as sufficiently expressed for a specific cell type. Results were normalized to the covered gene list per GO category of cortical neurons. Overall gene expression data of iPSC-derived cortical neurons shows slightly more covered genes in GO categories related to microtubule cytoskeleton organization, mRNA processing, RNA splicing and mRNA binding. PBMCs show lowest numbers of covered genes across nearly all selected categories.

To assess the purity of our bulk RNA-Seq data of generated iPSC-derived cortical neurons, the expression profiles were compared to publicly available single cell RNA sequencing data from human adult cortical samples (Darmanis et al., 2015; Figure 6B). The expression profile of generated iPSC-derived cortical neurons showed significantly high similarity to single cell expression patterns of cortical adult neurons (*p* < 10^–80^), while there was no or minor similarity to astrocytes, endothelial cells, microglia, oligodendrocytes and oligodendrocyte precursor cells (OPC) of the adult human cortex. PBMCs and lymphoblasts showed no similarity to any human cortical cell type in the same comparative analysis (Figure 6B).

## Discussion

This study presents a comparative data set of the expression of >160 MND-related genes in patient-derived primary biosamples (PBMCs and fibroblasts), immortalized cell lines (lymphoblasts, iPSCs) as well as cortical neurons reflecting the cell type affected by the disease. To model diseases with patient-derived cell types is especially of interest when analyzing (patho-)physiologically relevant processes in context of the patient’s mutation status. The endogenous expression of the causative gene is a pre-requisite for these studies.

**FIGURE 7:**
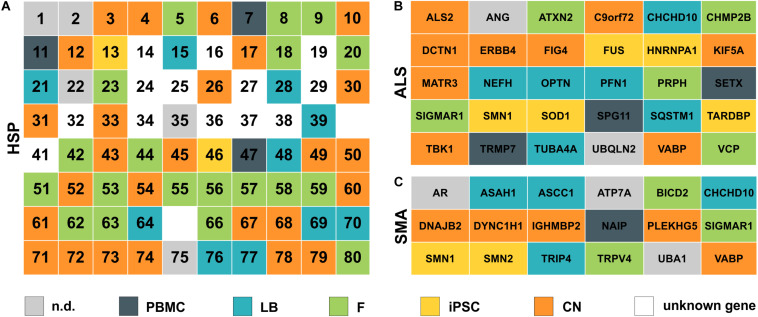
Simplified overview of analyzed HSP, ALS and SMA disease genes. **(A)** HSP genes are listed according to their SPG number (1–80). **(B)** ALS and **(C)** SMA genes are ordered alphabetically. Color defines the cell type with the highest expression for each gene. nd, not detectable; PBMC, peripheral blood mononuclear cells; LB, lymphoblasts; F, fibroblasts; iPSC, induced pluripotent stem cells; CN, iPSC-derived cortical neurons; unknown gene – only loci identified.

In general, the direct comparison of the expression data of two independent healthy individuals showed large similarity in expression profiles for a given cell type (e.g., fibroblasts) between individuals but a huge variability within the same individual across different cell types. Figures 2, 3 provide an overview on expression profiles to inform on suitable cell types that express the RNA of interest of >160 MND-related transcripts. Cells with highest endogenous expression are recommended for further studies to compare differences in protein expression between patients and controls and its pathophysiological relevance (Figure 7).

The highest expression of MND-related genes varied largely between the five cell types on analysis in this study. 8.3% of MND genes were most abundant in PBMCs, 15.5% in lymphoblasts, 24.4% in fibroblasts, 13.1% in iPSCs and 29.8% are expressed highest in CNs, while 8.9% of all MND-related genes could not be identified in any of the analyzed cell types. Comparable distributions could also be seen for all three groups of MND with CNs being the most commonly represented cell type (HSP: 40%, ALS: 30%, SMA: 28%). An overview of all HSP, SMA and ALS genes and the respective cell type with the highest expression is provided in Figure 7. This clearly shows that a general statement of an ideal cell type for disease modeling of MNDs is not valid. Nevertheless, iPSC-derived cortical neurons and/or fibroblasts are in most cases more suitable cell types compared to PBMCs or lymphoblasts.

We selected projection neurons of cortical layer V/VI for this study as they can be differentiated with high purity from iPSC and motor neurons constitute a specific subtype of projection neurons originating from cortical layer V/VI. Our differentiation protocol allowed for a reproducible generation of a largely homogeneous neuronal cell population with 100% of cells expressing ß-III-tubulin as neuronal marker and >85% of cells being positive for CTIP2 and/or TBR1 expression indicating cortical projection neurons of layer V/VI (Rehbach et al., 2019). This allows to perform bulk RNA-Seq analysis of neurons without cross-contamination of cells of glial origin as demonstrated by the non-detection of glial markers like GFAP as well as the comparative analysis to single cell RNA-Seq data of human cortical samples. Established motor neuron protocols do not reach such an extent of purity and represent lower (rather than upper) motor neurons that are not affected in HSP (reviewed in Sances et al., 2016).

The intuitive notion that the expression of MND genes is highest in cortical neurons as the primary affected cell type in HSP and ALS is only true in ∼30% of all MND genes. This may reflect that the function of a motor neuron and especially its extremely long axons require additional functionality of supporting cells including oligodendrocytes, microglia, astrocytes as well as peripheral Schwann cells and is further influenced by more general systemic metabolic pathways and changes. In extreme cases, especially when gene products play a role in general metabolic pathways like ARSI (SPG66) or CYP27A1 (CTX), the expression of the RNA is absent in iPSC-derived cortical neurons but is highest in fibroblasts or PBMCs (Figures 2, 3). In contrast, more easily available patient-derived cell types like fibroblasts and PBMCs do not express several relevant MND genes like SPG10 (KIF5A), SPG26 (B4GALNT1), SPG31 (REEP1), ERBB4 and PLEKHG5 and therefore disqualify as cellular models for pathogenic studies in these MNDs.

Studying the expression profiles by looking at specific clusters that share a similar cellular function as summarized by Blackstone (2018), some pathophysiologically related HSPs show similar expression profiles. HSPs associated with axonal transport deficits like SPG4 (SPAST), SPG10 (KIF5A), SPG30 (KIF1A), SPG31 (REEP1), and SPG72 (REEP2) share highest expression in cortical neurons as their primary site of action and are only low expressed or absent in all other analyzed cell types. These findings could further be validated by the proteomic analysis, where e.g., ATL1, SPAST, and REEP1 could exclusively be detected in CNs. Interestingly, some RNAs including PLP1 (SPG2), MAG (SPG75), and GFAP (Alexander disease) are expressed in none of the cell types studied here. These genes define subtypes of MNDs with primary myelin or glial dysfunction. For example PLP1 (proteolipid protein) is the primary constituent of myelin in the central nervous system (Diehl et al., 1986). In this specific subset of MND-related genes a suitable cell culture model probably requires different approaches and cell types like iPSC-derived oligodendrocytes, Schwann cells and/or astrocytes or even a co-culture or a 3-dimensional *in vitro* culture system to obtain meaningful and disease-relevant results (Shaltouki et al., 2013; reviewed in Douvaras et al., 2014; Kim et al., 2017; reviewed in: McComish and Caldwell, 2018).

In summary we provide a valuable dataset of more than 160 MND-related transcripts of different patient-derived biosamples, which can be employed to preselect a proper cellular model to potentially decipher molecular mechanism wit pathophysiological relevance and to screen for novel therapeutic targets in various types of MNDs.

## Data Availability Statement

Data can be found in NCBI using the accession number GSE157185.

## Ethics Statement

The studies involving human participants were reviewed and approved by Ethics Committee of the University of Tübingen (598/2011BO1). The patients/participants provided their written informed consent to participate in this study.

## Author Contributions

SH and LS: conceptualization. SH, SS, and JA: methodology. JA, AV, BM, and SO: software. SH, SS, JA, and AV: formal analysis. SH, SS, EH, PH, JA, YS, and AV: investigation. SO and BM: resources. SH: writing – original draft and visualization. SS, EH, PH, JA, AV, BM, SO, and LS: writing – review and editing. SH, SO, and LS: supervision. SH and LS: project administration. LS funding acquisition. All authors contributed to the article and approved the submitted version.

## Conflict of Interest

The authors declare that the research was conducted in the absence of any commercial or financial relationships that could be construed as a potential conflict of interest.
